# The Use of Bovine Colostrum in Sport and Exercise

**DOI:** 10.3390/nu13061789

**Published:** 2021-05-24

**Authors:** Glen Davison

**Affiliations:** School of Sport and Exercise Sciences, Division of Natural Sciences, University of Kent, Canterbury CT2 7PE, UK; g.davison@kent.ac.uk

**Keywords:** immunity, illness, upper respiratory infection, athlete, gut permeability, gastrointestinal, performance, training, intensified

## Abstract

There has been a great deal of interest in bovine colostrum within sports nutrition over the last 25 years. Studies have investigated the effects on body composition, physical performance, recovery, gut damage and permeability, immune function, and illness risk. This narrative review considers available evidence in each of these areas. Although some studies have shown protection against performance decrements caused by periods of intensified training, there is limited evidence for effects on body composition and physical performance. There is stronger evidence for benefit on gut permeability and damage markers and on immune function and illness risk, especially during periods of intensified training. The balance of available evidence for gut permeability and illness risk is positive, but further research is required to fully determine all mechanisms responsible for these effects. Early suggestions that supplementation with bovine colostrum products could increase systemic IGF-1 levels are not supported by the balance of available evidence examining a range of doses over both short- and long-term periods. Nevertheless, dose–response studies would be valuable for determining the minimum efficacious dose, although this is complicated by variability in bioactivity between products, making any dose–response findings applicable only to the specific products used in such studies.

## 1. Introduction

Bovine colostrum, its uses and potential health benefits are covered in the introductory article of this Special Issue by Playford and Weiser [1], which builds on previous work in this area [2]. Briefly, bovine colostrum (early milk) is the initial milk produced by cows, usually obtained within the first 48 h postpartum. Like ‘normal’ milk, it contains a rich source of nutrition, both in terms of macronutrients and micronutrients but is also abundant in bioactive components including immune, growth, and antimicrobial factors [1,2]. For a comprehensive overview of the main constituents of bovine colostrum and the factors that shape its quality, readers are referred to these reviews [1,2]. Briefly, compared to mature dairy milk, bovine colostrum contains around double the total solids, double the fat content, ~5-fold the protein content, and significantly higher content of most of the micronutrients. It also contains between 50-fold and 300-fold higher concentrations of immunoglobulins (IgG, IgA, IgM), up to 250-fold higher concentrations of lactoferrin, and up to double the content of other antimicrobial proteins and peptides (such as lactoperoxidase and lysozyme). It is also rich in cytokines, immune regulatory factors, and growth factors. Most likely, it is the relative concentration and bioactivity of these components that ultimately affects the quality of bovine colostrum and the extent to which it influences each outcome measure in humans (for example, low molecular weight components that are biologically available after digestion may be responsible for systemic immune-related effects, whereas growth factors may have local effects within the gut). A number of studies have sought to determine whether daily supplementation with bovine colostrum provides any benefit to exercise performance or has other direct or indirect benefits for those engaged in regular physical training, such as athletes. This includes potential direct effects on physical performance and/or recovery or indirect effects via improving health outcomes, protecting the gut against stressors/insult, maintaining optimal immune function, and reducing illness risk, each of which is discussed further in this review. It is important to note that there are many factors which influence the quality (and bioactivity) of colostrum, including cow’s health status, amount of colostrum produced, calving season, breed, age, production system, length of non-lactating period, prepartum milking, time delay between parturition and first milking, and thermal processing methods (indeed, some pasteurization methods have been shown to reduce IgG content by almost 60% [2]). Readers are directed to the review of McGrath et al. [2] for further detail on such factors. Unfortunately, information on these factors is seldom reported in studies evaluating the effects of colostrum. This is mainly due to the fact that the vast majority of commercially available bovine colostrum, which is used in virtually all clinical trials, is the composite of hundreds to thousands of different cattle. To overcome issues of variability, the most pragmatic approach may be for researchers to report the bioactivity level of the colostrum used in their research.

## 2. Studies Related to Physical Performance

### 2.1. Body Composition and Strength

Some early studies suggested that colostrum may enhance body composition by promoting muscle gain and fat loss. In conjunction with resistance training, there is some evidence that, compared with placebo, bovine colostrum can promote greater gains in muscle mass, strength, and losses of body fat [3,4], although other studies have shown no differences [5,6]. A partial explanation for this variability may be the fact that many studies use a placebo that is not matched for energy and/or protein content. This and/or other differences in nutritional composition could therefore be a contributing factor in the enhanced outcomes reported in some studies. Indeed, colostrum contains many micronutrients (in much greater abundance than whey, which is commonly used as the placebo), which could have played a contributing role in enhanced adaptation to strenuous training regimes. In particular, colostrum supplements typically contain more calcium, which could influence lipid metabolism and body composition in athletes. Supplemental calcium between 600–1000 mg/day has been shown to increase fat loss or prevent fat gain during periods of positive energy balance [7], so the additional calcium that subjects would have been consuming (250–750 mg per day) in the colostrum groups may confound such findings. More research is required, with better matched placebo and control conditions, to more thoroughly determine the effects of colostrum, per se, on body composition responses to training in athletes.

### 2.2. High-Intensity and Intermittent Exercise Performance

Fatigue in high-intensity exercise (e.g., intensities that cause exhaustion in 1–10 min) is related to limited capacity and rate of anaerobic glycolysis, metabolic by-product accumulation, and muscular acidosis via increased hydrogen ions (H+) [8,9]. Likewise, activities such as sprinting, or repeated sprints with short recovery intervals, are limited by multiple factors including the decline in muscle ATP and phosphagen pools and the accumulation of metabolic by-products (including H+) [9,10]. A number of studies have demonstrated that this type of performance can be enhanced by increasing buffering capacity via nutritional means (either intracellular buffer capacity to sequester H+ or extracellular buffer capacity allowing greater efflux of H+ out of the muscle cells) [9,10]. Bovine colostrum supplementation has been suggested, in some studies, to exert effects via such a mechanism. Brinkworth et al. [11] showed that 60 g/d bovine colostrum supplementation, during a 9-week training programme in female rowers, increased blood buffer capacity compared with the placebo (whey protein). However, training is a potent stimulus for increasing the body’s buffering capacity, so it is not clear whether this was a direct effect of bovine colostrum or an indirect effect via promoting a more favourable training response. Furthermore, there was no difference in performance in 2 repeated incremental rowing tests, separated by 15 min of recovery. However, as mentioned above, increasing buffer capacity is most likely to influence exercise that normally causes exhaustion within 10 min, whereas total exercise time was 16 min in the incremental test. There is convincing evidence for performance benefits in activities up to 10 min with other nutritional interventions that increase buffer capacity [8,9], so if bovine colostrum does increase buffer capacity, then it could potentially improve performance via this mechanism. It is worthy of note, however, that buffering capacity can be increased acutely with sodium bicarbonate ingestion and/or in the longer-term with beta-alanine. Hence, more effective nutritional strategies are available for athletes seeking to benefit from enhanced buffering capacity.

Kotsis et al. [12] investigated the effect of 6 weeks of low dose (3.2 g/d) bovine colostrum supplementation on performance recovery following an intermittent exercise task designed to simulate the physiological demands of soccer. Colostrum attenuated inflammatory and muscle-damage-related markers induced by their protocol and speeded the recovery of explosive power (assessed via squat jump performance). This could have implications for adaptation and chronic improvements in performance in response to training for intermittent sports. 

### 2.3. Bovine Colostrum Supplementation and Endurance Performance

In addition to the intermittent sport (soccer) study mentioned above [12], there is evidence for enhanced recovery from other forms of demanding exercise (and improved subsequent performance) with colostrum supplementation. Buckley et al. [13] provided participants with daily supplements (60 g/day) of powdered bovine colostrum or placebo (whey protein) during an 8-week intervention period. Participants trained 3 times per week (45 min of running per session) and exercise capacity was assessed, pre-intervention (baseline) and after 4 and 8 weeks. The assessment required participants to perform an incremental treadmill run to volitional exhaustion, rest for 20 min, and repeat. There was no difference in running ‘performance’ between the colostrum and placebo groups at baseline or week 4, but by week 8 the colostrum group showed greater improvements in distance covered in the second treadmill test. Mechanistic markers were not assessed but possible factors contributing to this outcome include improved metabolic buffering capacity or other systemic factors contributing to enhanced physiological training adaptation, enhanced immune function/reduced illness (resulting in fewer days of missed training during the study period, see Immunity section below), and greater adherence to prescribed training. Shing and colleagues [14] studied a group of cyclists on daily supplementation (10 g/day) with a concentrated bovine colostrum powder for 6 weeks. They observed that the decrease in time-trial performance that was caused by a 5-day period of intensified training in the sixth week was prevented with colostrum (although there was no benefit to time-trial performance during normal training). Additionally, Coombes et al. [15] assessed endurance performance in cyclists before and after 8 weeks of supplementation with placebo, 20 g, or 60 g per day of bovine colostrum. They observed that performance improved more in the colostrum groups, compared with the placebo group, but there was no difference between the 2 colostrum doses. As only 2 doses were used (20 g and 60 g/day), this is not dose–response study per se, but it does suggest that there is little value (in the context of this study) to be gained from doses in excess of 20 g/day. Further research is required, with a full range of doses up to 20 g/day (dose–response) to determine the optimal dose, although this is likely to vary between individuals and even within individuals under different conditions (e.g., intensified training vs. normal training periods). It will likely also vary between different products, as bioactivity varies between these (see below). In summary, there is some evidence from well-conducted placebo-controlled experimental trials that bovine colostrum supplementation may be beneficial to endurance performance, especially during periods of intensified training (a strategy sometimes employed by endurance athletes).

## 3. Gastrointestinal Integrity

Many stressors, such as heat stress, certain types of medication, and oxidative stress are known to cause disturbance to gut function and integrity. A number of these stressors may be induced by strenuous exercise (e.g., heat stress, oxidative stress), which has been shown to increase gastrointestinal permeability. This increases potential translocation of luminal toxins and bacteria into the systemic circulation (endotoxaemia). This may place additional stress on the immune system, which must then deal with these challenges, and this may be one factor contributing to exercise-induced immunodepression (see next section). However, increased intestinal permeability may also contribute to increased incidences of some acute gastrointestinal complaints and symptoms in athletes, such as stomach cramps, nausea and dizziness, and diarrhoea [16]. A high prevalence of such symptoms has been noted in some groups of athletes after strenuous competitive events (e.g., up to 50% incidence after marathon and ultra-endurance events, and even > 90% incidence during and after an Ironman Triathlon event) [16,17]. Such symptoms have potential negative effects on performance if incurred during an event, or if they develop afterwards, may impede subsequent recovery, dietary intake, nutrient absorption, training, or performance. Under extreme circumstances severe endotoxaemia can be a contributing factor to acute inflammation, sepsis, shock, and multiple organ failure, which could be fatal. Indeed, it has been suggested that damage to the gut can be a contributing factor in exertional heat stroke [18]. Recently, Walter and colleagues [19] demonstrated a substantially higher systemic concentration of a marker of enterocyte cell damage (intestinal fatty acid binding protein, iFABP) in athletes who collapsed after a marathon compared with those who did not, providing further evidence for the potential role and importance of the gut barrier in this pathway. 

**Figure 1:**
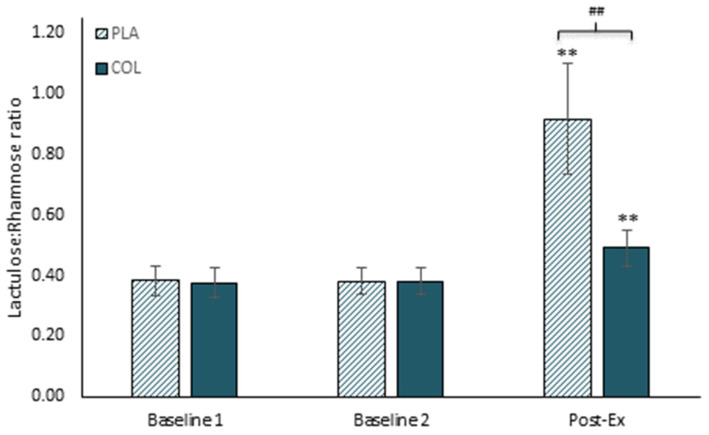
Gut permeability from Marchbank et al. [20] by 5 h urine collection. Dual sugar probe test: 5 h urine collection after ingestion of lactulose and rhamnose. Baseline measurements taken at rest and Post-Ex (post-exercise) measures taken after 20 min running at 80% VO_2_max following 2 weeks of placebo (PLA) or colostrum (COL) supplementation. ** *p* < 0.01 compared with baseline (rest); ## *p* < 0.01 colostrum compared with placebo.

Supplementation with bovine colostrum has been shown to be beneficial in maintaining gastrointestinal integrity and permeability. For example, Marchbank et al. [20] conducted a randomised placebo-controlled crossover trial to determine the effects of bovine colostrum supplementation on exercise-induced changes in gut permeability (see Figure 1). Participants ran for 20 min on a treadmill under controlled laboratory conditions (causing a core body temperature increase of between 1–2 °C) after 2 weeks supplementation with 20 g/day bovine colostrum or a protein and energy-matched milk protein concentrate placebo. Running caused a significant increase in gastrointestinal permeability in the placebo trial, but this was almost completely blunted in the colostrum trial. This finding was replicated in a follow-up study by Davison et al. [21]. Similar effects were also observed by March et al. [22], with 2 weeks of 20 g/day bovine colostrum supplementation blunting the exercise-induced increase of iFABP (as a marker of damage to gut epithelial cells, which may be one of the mechanisms contributing to increased permeability). March and colleagues [23] later examined the effects of this supplementation strategy on exercise in the heat, which is known to exert greater thermal stress and to induce greater damage and increases in gastrointestinal permeability. In this study, participants were required to exercise for 60 min in an environmental chamber maintained at 30 °C and 60% relative humidity. This caused significant increases in iFABP that were blunted by colostrum. They also observed lower overall systemic levels of bacterial DNA (independent of the exercise effects), although permeability was not directly measured in this study. Other studies have also reported beneficial effects of bovine colostrum on gastrointestinal permeability in athletes, even with comparatively low doses (e.g., 500 mg/d in [24]). 

Several studies have observed null effects on gastrointestinal permeability or damage markers. McKenna et al. [25] observed no difference between placebo and colostrum on iFABP responses to exercise in the heat. These authors proposed that their lack of an observed effect may be related to core temperature being elevated above 39 °C, suggesting the extent of damage caused by this was too great for colostrum to alleviate. However, core temperatures were in excess of 39 °C in the March et al. [23] study, in which beneficial effects were observed for this marker. It is likely, therefore, that the discrepant findings of McKenna et al. [25] are not explained by this theory. Of note, in the McKenna study [25] there were variable running times between the two conditions (placebo and colostrum), and this could have confounded their findings. Morrison et al. [26] also observed no benefit of colostrum on the exercise-induced increase in plasma iFABP. This study involved alternating periods of running and cycling (75 min total exercise time) at 30 °C, 50% relative humidity. Morrison et al. [26] did attempt to apply permeability assessment (urinary lactulose: rhamnose) but their methods lacked the sensitivity to detect the requisite sugar probes. They also based their colostrum dose on a previous rat model, selecting 1.7 g/kg/d. This is a very high dose (e.g., ~120 g/day for an average adult) compared with the 10–20 g used in most human trials. It is possible that such a large dose could also have some negative effects which may counteract any potential benefits (e.g., this would mean the consumption of an additional ~70 g protein and ~35 g lactose per day, which could have negative effects on the gastrointestinal system). Other potential explanations for discrepant findings could lie in the products used. Research has shown differences in bioactivity between different colostrum products [27,28], and this could have implications for the effects observed in vivo, in exercise studies. Of note, the products used in the studies from our group (where beneficial effects have been observed, [20,21,22,23]) show high levels of bioactivity in such in vitro tests, whereas this is not always known for other products, such as those used in Morrison et al. [26] or McKenna et al. [25]. As suggested by Playford et al. [27], it may be appropriate for researchers to report the bioactivity level of the colostrum being used in their publications.

In summary, bovine colostrum supplementation may be beneficial in preventing exercise-induced increases in gut permeability, and there is some evidence that this may be beneficial to athletes (e.g., by indirectly impacting upon training and performance), especially in those required to exercise or compete in hot environments.

### Bovine Colostrum in Combination with Other Nutrients

As noted by Playford and Weiser [1], some commercial bovine colostrum products also contain other components (e.g., proteins, egg, carbohydrates, vitamins, probiotics, and plant polyphenols) that may have additional biological effects in vivo. There is limited research, however, that has examined the relative contribution of each component and whether they exert additive or synergistic effects with bovine colostrum. One exception to this is the finding of our group, using a randomised crossover design, showing that bovine colostrum and zinc carnosine given together had additional benefits over using either product alone: this included reducing heat-stress-induced perturbations on cell lines in vitro, and reducing exercise-induced increases in gut permeability [21]. Although not an exercise study, Playford and Marchbank [29] also demonstrated that the zinc carnosine macromolecule provides additional benefit over using equimolar carnosine and zinc sulphate in combination in preserving gut integrity in mice exposed to gut ischaemia–reperfusion stress, providing further support for the mechanisms we observed in the exercise study. The potential synergy we observed could be important, as it may allow the same or greater effects to be obtained with smaller doses, which is another factor to be aware of when considering dose–response studies.

## 4. Immunity and Infection in Athletes

Upper respiratory tract infections (URTI) are among the most frequent presentations to physicians [30]. Although typically minor in nature, such illnesses can have significant economic and social impacts (e.g., absence from work; healthcare costs; increased morbidity; reduced feelings of well-being, health, and quality of life; and reduced social interaction). URTI are also among the most common type of infection in athletic populations [31,32,33], shown to be highly prevalent within athletes at clinics in both the summer and winter Olympic Games (e.g., [34,35,36,37]). It is generally accepted that moderate amounts of exercise improve immune system functions and hence reduce susceptibility to infection. In contrast, athletes engaged in regular prolonged and/or intensive training appear to have a higher than ‘normal’ incidence of URTI. Such illness may have impacts on performance capability, either directly (i.e., if suffered shortly before or during competition) or indirectly (i.e., affecting training and/or physiological adaptations). The reasons an athlete might demonstrate increased susceptibility to URTI are complex, including factors that influence the athlete’s host defence (immune functions) and of course, environmental risks and exposure factors (i.e., coming into contact with pathogens). In addition to the physiological stress of strenuous exercise, other influences include psychological stress (which may be related to competition and/or general life stressors), environmental conditions, sleep, travel, and nutritional status. Although the relative importance and contribution of each factor has been heavily debated in recent years (e.g., exercise-induced immune changes vs. exposure factors [38]), it is likely an oversimplification to consider any single factor in isolation. However, it is quite clear that certain groups of athletes have a higher likelihood of presenting with URTI symptoms in general and/or at certain times (e.g., periods of intensified training, after attending training camps, and in the build up to and during major competitions) [34,35,36,37,39,40]. For this reason, any intervention that can mitigate this has potential value to such individuals. Research in this area has followed two general approaches (either alone or in combination). One approach is to focus on immune function (and other related markers), and the other is to focus on the clinically relevant endpoint, URTI or symptoms thereof.

Common immune markers include peripheral blood immune cell numbers and functions, mucosal immune markers, and systemic concentrations of regulatory factors such as cytokines and hormones. Such markers may be valuable for providing mechanistic insight, but in isolation they may lack clinical or biological relevance. Measures of in vivo immune function are considered more ‘clinically relevant’, as they provide information on the ability of the whole integrated system to mount a co-ordinated response and defend against infection [41,42]. Actual presentation with URTI episodes, as the clinically relevant endpoint, is obviously the most desirable outcome measure. However, many of the studies that include this outcome utilise a self-report method that employs an illness questionnaire or log. Although this approach often utilises validated questionnaires, some questions have been raised regarding the validity of self-reported URTI episodes [41,42,43,44,45,46]. Few studies have included laboratory confirmation of infectious cause [37,45,47], and some report discrepancies between physician and laboratory diagnosed URTI [43]. However, it is important to note that a negative result in the laboratory screening test does not necessarily mean that the athlete was not ill or that a physician was incorrect in their diagnosis—it could be that the laboratory diagnosis method was not able to detect the pathogen causing the infection. Indeed, the typical procedures (e.g., polymerase chain reaction, PCR) are targeted to specific pathogens and may be ineffective at identifying new pathogens or novel variants of existing ones [48]. Indeed, this point was demonstrated by Kustin et al. [48], whereby 54 of 300 samples from patients presenting with URTI symptoms were negative using traditional screening methods (i.e., PCR pathogen panels). However, using a next generation sequencing (NGS) approach, they were able to identify a URTI pathogen in all 54 samples that would have been otherwise classified as negative via the ‘traditional’ methods. This suggests that some of the apparent discrepancies between laboratory detection of pathogens and self-report and/or physician diagnosis in studies such as Spence et al. [45] and Cox et al. [43] could be explained by the methods employed to screen for the pathogens, or the specific pathogens targeted. Moreover, in some respects if an intervention can reduce URTI symptoms (regardless of the cause), this will be of benefit to the athlete, although the mechanism by which they exert such effects, and their efficacy, will be influenced by the cause (i.e., infective or non-infective), so it remains important to be mindful of the potential limitations with the self-report method. For this reason, studies will often employ a combination of methods, with high value markers known to be important to host defence in combination with monitoring of URTI symptoms.

Bovine colostrum has received considerable attention within Exercise Nutrition and Immunology research. In vitro studies in which colostrum or isolated fractions thereof are added to incubation media generally show an enhancement or priming of ex vivo immune cell functions (including oxidative burst and degranulation, cytokine production, and other effector functions in response to stimulation [49,50,51,52,53,54]). It has been suggested that such effects are attributable to the low molecular weight (MW) components (<10 kDa), although further investigation is required to explore this in vivo [55].

Benefits have also been observed in studies in which blood samples are obtained from participants after daily supplementation. For example, Jensen et al. [56] observed an increase in peripheral blood neutrophil phagocytic activity 1 and 2 h after the consumption of a low MW fraction from colostrum, compared with placebo. Mucosal immunity has also been commonly assessed, most typically via saliva IgA and/or antimicrobial proteins. Crooks et al. [57] showed 12-weeks bovine colostrum supplementation increased resting saliva secretary IgA (s-IgA) by 79% in distance runners, and Mero et al. [58] observed a 33% increase in saliva s-IgA after 2 weeks of supplementation. Davison and Diment [59] observed that 4 weeks of daily bovine colostrum supplementation (20 g per day) prevented the prolonged exercise-induced decrease of salivary lysozyme and speeded the recovery of blood neutrophil functions post-exercise. However, Davison and Diment [59] and Jones et al. [60] found no effects (after 4 weeks or 12 weeks supplementation, respectively) in resting neutrophil functions in physically active men. Davison and Diment [59] did report a trend for resting (stimulated) neutrophil degranulation to be enhanced, however. Additionally, although some studies have observed increases in resting saliva s-IgA levels after colostrum supplementation, others have not [57,59,60,61,62,63,64]. Shing et al. [64] investigated the effects of bovine colostrum on salivary s-IgA in addition to selected markers of innate immunity. They observed some beneficial effects on some immune measures but not salivary s-IgA. Overall, the findings on ex vivo markers in blood and/or saliva are quite varied. A recent meta-analysis [65] concluded that colostrum has little to no impact on such ex vivo immune or immune-related markers. However, as previously mentioned, in vitro (and ex vivo) markers of immune function may provide some mechanistic insight to accompany clinically relevant markers and outcomes but are low value in their own right in the context of URTI risk. On the other hand, in vivo and clinically relevant markers are most useful [41,42]. It is recognised that challenge-type tests, including vaccine-specific antibody production, are the most valuable in vivo markers and measures, considering biological relevance and clinical significance [41,42]. He et al. [66] reported that colostrum supplementation may enhance the production of specific IgA following an oral Salmonella vaccine, although this did not reach statistical significance. Wolvers et al. [67] examined the effects of BC supplementation (1.2 g/day) on responses to tetanus and typhoid vaccines. They also observed trends for augmented antibody responses (e.g., IgG responses—tetanus: control, week 8 = 17-fold increase; week 10 = 25-fold increase; BC, week 8 = 26-fold increase; week 10 = 31-fold increase; typhoid: control, week 8 = 3.7-fold increase; week 10 = 3.2-fold increase; BC, week 8 = 4.2-fold increase; week 10 = 3.6-fold increase). As with He et al. [66] they did not observe statistical significance, but the sample size was relatively small. The dose of colostrum (1.2 g) was also modest by comparison with the exercise studies in which 10–20 g/day are typically used. Satyaraj et al. [68] found that bovine colostrum enhanced the response to the canine distemper virus (CDV) vaccine in sled dogs (working animals with very high daily activity levels). Hence, despite the lack of significance in the human trials [66,67], the vaccine response model should be further explored in athletes (possibly with doses up to 20 g/d) to examine the utility of colostrum in the context of augmenting vaccine responses in highly active humans.

**Figure 2:**
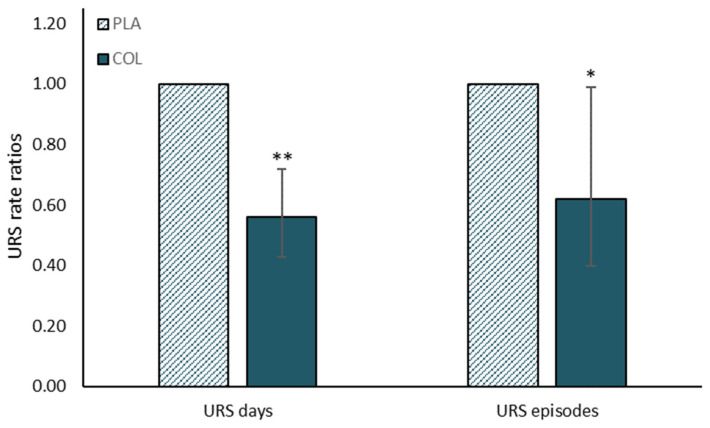
Summary of findings from Jones et al. [71] meta-analysis on effects of bovine colostrum supplementation on upper respiratory symptoms during exercise training. URS days, self-reported total days with symptoms; URS episodes, number of episodes during monitoring period. PLA bars show reference rate ratio of 1.0, and COL bars show rate ratio relative to PLA and 95% confidence intervals. * *p* < 0.05, ** *p* < 0.01 compared with PLA.

Cesarone et al. [69] examined the effects of bovine colostrum in healthy participants and high-risk cardiovascular patients, reporting reductions in the relative risk of influenza episodes (self-reported). Numerous exercise studies have also shown beneficial effects on high value immune markers and/or URTI risk (e.g., [60,70]) with the effects on infection more recently the subject of a meta-analysis by Jones et al. [71]. This analysis concluded that bovine colostrum supplementation reduced the incidence of URTI symptom reporting and the duration/number of days for which an episode persisted in individuals engaged in regular exercise training (see Figure 2). Most recently, Jones et al. [72] used the in vivo model of experimental contact hypersensitivity (CHS), revealing that 4 weeks of daily supplementation (20 g/day) enhanced the sensitivity to this antigenic challenge (compared with placebo) following 2 h running exercise. This is a well-established method of assessing in vivo immunity in response to exercise [73,74,75], in which significant reductions in CHS sensitivity are observed following prolonged exercise (2 h). Jones et al. [72] found that total CHS response was not affected by bovine colostrum, but sensitivity was greater following exercise in the colostrum vs. placebo group (i.e., colostrum protected against exercise-induced reductions in sensitivity to antigenic challenge), suggesting that this may be most relevant component of CHS response in relation to in vivo host defence and URTI risk. This study, therefore, provides evidence for an enhancement of in vivo immune function that may explain the reduced URTI observed in previous studies [71]. In summary, there is evidence that daily bovine colostrum supplementation, for 2–12 weeks, can have beneficial effects on clinically relevant immune functions and reduce the chances of suffering upper respiratory tract infections or symptoms. This may be most apparent in individuals subject to additional immunodepressive stressors (e.g., during intensified periods of training). Readers interested in the more general effects of bovine colostrum on immune function are also referred to the accompanying article in this special issue by Ghosh et al.

## 5. Risks and Controversies?

**Figure 3:**
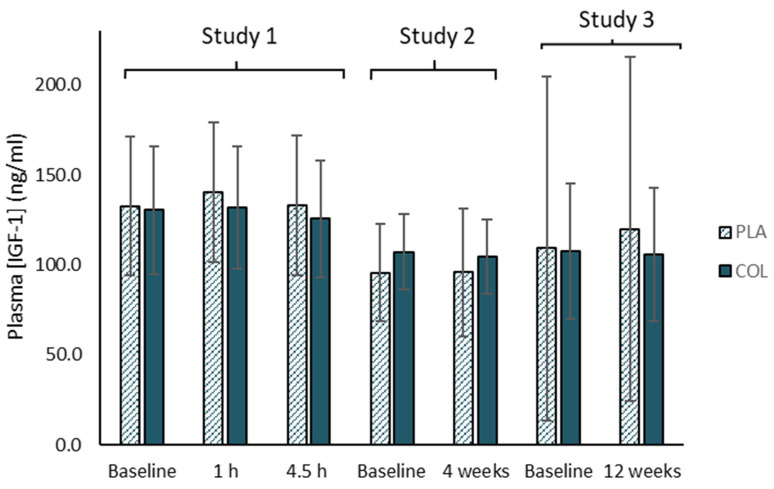
Summary of results from Davison et al. [76] showing no effects of acute (1–4.5 h post-ingestion: study 1) and chronic daily (4 weeks: Study 2; and 12 weeks: Study 3) bovine colostrum supplementation on IGF-1 levels.

In 2013, the World Anti-Doping Agency (WADA) first published a statement that bovine colostrum contains ‘high’ levels of IGF-1 and other growth factors which could influence the outcome of a doping test, and for this reason they do not recommend its consumption. However, bovine colostrum is not prohibited, meaning many athletes and active individuals may continue to use bovine colostrum. The WADA statement seems to be based on only two studies reporting apparent increases in IGF-1 with colostrum supplementation. However, closer examination of these studies and their results reveal some problems with such an interpretation. These 2 studies are very much against the balance of evidence, however, with a large number of other studies showing no such increase (e.g., [6,15,76,77], e.g., see Figure 3). The discrepancies may be explained by methodological issues arising from inappropriate statistical comparisons in the first study [78] and/or the composition of the placebo [58]. Mero et al. [78] reported relative increases, but these were confounded by vast differences in the baseline levels. Indeed, the baseline level in the placebo trial was higher than the highest level attained after colostrum supplementation, so the interpretation of an apparent increase in the colostrum trial (to levels lower than the baseline placebo levels) is a questionable one. Mero et al. [58] observed an increase in IGF-1, but they used maltodextrin as the placebo (so it was not protein-matched to the colostrum supplement), and it is known that protein intake per se stimulates endogenous IGF-1 synthesis and that higher protein intakes are associated with higher systemic IGF-1 levels [79,80]. Most research, however, uses a placebo that also contains protein or is matched in macronutrient content to the colostrum product and has observed no differences in IGF-1. In further support that oral IGF does not increase serum IGF levels, Mero et al. [58] also examined the effect of orally administered 125I labelled IGF-I and showed that it was digested and fragmented into inactive forms.

Many studies have shown no effects of oral consumption of bovine colostrum on systemic IGF-1 levels when examining a range of doses taken over both short term (1–4.5 h post-ingestion) and longer term periods (4–12 weeks of daily supplementation), especially when an appropriate placebo is used—i.e., isocaloric and iso-macronutrient/protein content [6,76,77,81] (e.g., see Figure 3), because it is broken down during digestion [81]. Indeed, Kuipers et al. [81] found no effects of bovine colostrum on drug screening results carried out by an IOC accredited laboratory. Jones et al. [60] also undertook a comprehensive metabolomics analysis on the effects of 12-weeks of daily BC supplementation (20 g/day), and none of the changes in serum metabolites reported in that study suggested anything that may interfere with a doping test. Furthermore, when we have measured IGF-1 in bovine colostrum and other dairy products in our lab, we have found ~4-fold higher levels in colostrum vs. whey protein and ~64-fold higher in colostrum vs. normal dairy milk. However, when expressing these relative to typical servings or portions (e.g., 10 g powder products vs. 200 mL glass of milk), the colostrum would contain ~3.2-fold more IGF-1 (and 40 g of whey protein would provide this amount). The WADA statement does not seem to reconcile well, therefore, with the fact that athletes may regularly consume other dairy products, such as whey protein and milk (alone or in combination) containing significantly more IGF-1 than would be contained in colostrum supplements, yet no similar statements have been issued for these other products. Nevertheless, athletes should be mindful of this statement in relation to colostrum and the implications of this within their overall risk–benefit considerations in general. Again, dose–response studies may be informative here to determine the minimal dose required for specific products to elicit effects. 

## 6. Summary

In summary, there is some positive evidence for beneficial effects of bovine colostrum on body composition and physical performance (including recovery from demanding exercise). However, there are few studies, and sometimes potential confounding factors were not well controlled. The evidence base for this therefore remains minimal at present. The balance of available evidence is strong for beneficial effects in protecting against exercise-induced increases in gut permeability. There are mixed findings for in vitro and ex vivo immune markers, but the weight of evidence is strong for a benefit on more clinically relevant markers (including in vivo challenge-type measures and clinically relevant endpoints such as URTI reporting). Further research is required to better determine dose–response profiles, although this is complicated by large variations in bioactivity between different products. Such dose–response studies would be valuable for determining the minimum efficacious dose, although due to variability between products, such findings will be applicable only to the specific products used in these studies. Further studies exploring the mechanisms underpinning the efficacy of supplementation will also be valuable.

## Data Availability

Not applicable.
